# Reimbursement of Severe Trauma Care Under the SwissDRG Prospective Payment System: 11-Year Evolution of Cost-Reimbursement Adequacy

**DOI:** 10.3390/healthcare14213761

**Published:** 2026-11-04

**Authors:** Oskar Bougaud, Giulio Cisco, Mohamed Jalal Rahmani, Tobias Zingg, Charles André Vogel, Fabian Grass, Fabio Agri

**Affiliations:** 1 Faculty of Biology and Medicine, University of Lausanne (UNIL), 1015 Lausanne, Switzerland; oskar.bougaud@unil.ch; 2 Center for Primary Care and Public Health (Unisanté), University of Lausanne, 1010 Lausanne, Switzerland; giulio.cisco@unisante.ch; 3 Department of Administration and Finance, Lausanne University Hospital CHUV, University of Lausanne (UNIL), 1011 Lausanne, Switzerland; mohamed-jalal.rahmani@chuv.ch (M.J.R.);; 4 Department of Visceral Surgery, Lausanne University Hospital CHUV, University of Lausanne (UNIL), 1011 Lausanne, Switzerland; tobias.zingg@chuv.ch (T.Z.); fabian.grass@chuv.ch (F.G.)

**Keywords:** prospective payment system, SwissDRG, polytrauma patients, cost-reimbursement analysis, deficit predictors

## Abstract

**Highlights:**

**What are the main findings?**

• In Switzerland, inpatient prospective payment remains insufficient for severe polytrauma care despite >10 years of adjustments and predicted cost coverage.
• Predictive modeling identified intensive care unit (ICU)-related DRGs and polytrauma-specific DRGs as key deficit drivers.

**What are the implications of the main findings?**

• Substantial historical losses remain unrecovered, threatening the financial sustainability of highly specialized trauma services.
• Reimbursement should better account for deficit predictors and the resource intensity of complex trauma care.

**Abstract:**

**Background/Objectives**: The Swiss Diagnosis-Related Groups (SwissDRG) system, introduced in 2012, aimed to enhance cost transparency and reimbursement efficiency. However, trauma centers treating severely injured patients face financial challenges due to complex care needs and difficulties in estimating appropriate cost weights. This study assessed the adaptability of the SwissDRG prospective payment system since its implementation and identified key drivers of financial deficits in polytrauma care. **Methods**: We conducted a retrospective cohort study at Lausanne University Hospital, including 382 adult polytrauma patients with an Injury Severity Score ≥ 16, treated from 2012–2019. Reimbursement was evaluated using three methods: MOOS, which grouped the entire population using tariffs from the first three years of SwissDRG implementation; ACTUAL, which compared costs and reimbursements per patient and per year; and RECODED, which simulated updated DRG classifications over an extended 11-year period, up to 2023. Statistical modeling identified the main drivers of financial deficits. **Results**: The MOOS method showed near-full cost coverage by 2015. The ACTUAL method revealed an average loss of CHF −2751 per patient and a cumulative deficit of CHF −1.05 million over 8 years, with intensive care unit (ICU)-related and polytrauma-specific DRGs as main contributors. In highly complex cases (ISS ≥ 20; 273/382 [71.5%]) mean losses reached CHF −3588. The RECODED method found full cost coverage only in 2021, despite substantial cumulative deficits needing decades of surplus to offset. **Conclusions**: Current SwissDRG reimbursement remains inadequate for complex trauma care. A more tailored model reflecting ICU costs, outlier penalties, and care complexity is urgently needed to sustain high-quality trauma services in Switzerland.

## 1. Introduction

In response to rising healthcare costs and growing demands for greater reimbursement transparency, Switzerland introduced the Swiss Diagnosis-Related Groups (SwissDRG) system in 2012, replacing the previous daily lump-sum payment model with a prospective payment system (PPS) [1]. The SwissDRG system classifies inpatient hospital stays into Diagnosis-Related Groups (DRGs) based on diagnostic codes (ICD-10-GM), procedure codes (CHOP), and demographic variables such as age, sex, and type of admission. Each DRG is assigned a cost weight (CW), which reflects the relative resource use. Reimbursement is calculated by multiplying the CW by a hospital-specific base rate (BR), a fixed monetary conversion factor expressed in Swiss francs (CHF). In addition, where applicable, supplementary payments defined in the SwissDRG catalog are added directly to the total reimbursement amount [2]. This system was designed to standardize reimbursements across the country, enhance the efficiency of hospital resource utilization and cost management, and facilitate benchmarking of inpatient care quality and outcomes [3].

For each DRG, national CWs are calculated based on the average expenditures of hospitalized patients assigned to the same DRG, using data from a preceding billing period [4]. Nonetheless, the accurate estimation of CWs for rare DRGs remains challenging, given that these calculations rely solely on the costs associated with inlier cases [5].

Timely and appropriate adjustments to SwissDRG tariffs are essential to align reimbursement with evolving clinical practices and the integration of new medical technologies. While recent policy revisions seek to address the underfunding of complex cases, hospitals managing patients with severe injuries may nonetheless continue to face financial constraints that hinder the delivery of high-quality care [6].

These challenges are not unique to Switzerland. Across European case-based payment systems, adequately reimbursing rare, highly complex patients remains challenging, particularly when resource use exceeds the levels reflected in standard DRG payments [7]. Evidence from Germany has specifically documented discrepancies between reimbursement and hospital costs for patients with severe polytrauma [8]. In Switzerland, a prior study clearly underscored the challenges associated with the initial reimbursement of polytrauma cases following the introduction of the Swiss PPS. It demonstrated that cost-neutral care was not achievable under the first three versions of SwissDRG, with mean financial deficits per patient of CHF −12,065 in 2012 (version 1.0), CHF −6977 in 2013 (version 2.0), and CHF −2902 in 2014 (version 3.0) [9].

Specialized care for severely injured patients is both costly and resource-intensive, necessitating meticulous planning to ensure continuous access to specialized expertise. In 2010, aiming to enhance efficiency and centralize resources, the Swiss government classified trauma care under highly specialized medicine (HSM) and designated 12 official trauma centers (TCs). These centers underwent reassessment in 2015 and 2023, informed by data from the newly established Swiss Trauma Registry, which initiated mandatory data collection on 1 January 2015 [10,11].

As a result, in Switzerland, severely injured polytrauma patients are referred to a limited number of TCs. These tertiary care institutions are thus disproportionately exposed to rare but highly complex cases, increasing the risk of inadequate cost coverage and potentially contributing to their negative annual financial balance [12,13,14].

With a population of 9 million and an incidence of 0.2–0.3‰, the polytrauma CW in Switzerland is based on fewer than 3000 cases annually [15]. This limited dataset poses a significant challenge for ensuring accurate reimbursement, particularly given the substantial cost variability associated with these complex cases [16].

Nonetheless, the PPS is expected to adjust progressively over time to achieve fair reimbursement levels, approaching full cost coverage for this patient population. Based on earlier projections, this milestone was anticipated to be reached by 2015 [9].

More than a decade after the implementation of the SwissDRG prospective payment system (PPS), this study evaluated the long-term impact and adaptability of polytrauma reimbursement. Specifically, it examined whether, after years of tariff adjustments, the system accurately reflects the actual costs of care for severely injured patients treated at a tertiary medical center. Furthermore, the study aimed to identify the main drivers of hospital deficits by applying a systematic variable selection approach to isolate the most relevant explanatory factors and to propose recommendations for optimizing reimbursement policies. By combining an extended observation period with retrospective recoding and a systematic predictive modeling approach, this study aimed to provide a comprehensive assessment of the long-term adequacy of SwissDRG reimbursement for severe polytrauma care. It also offers a novel framework for examining the factors associated with financial deficits and reassessing previous projections regarding the timeline for achieving financial break-even.

## 2. Materials and Methods

This retrospective, single-center cohort study focuses on polytrauma patients treated at Lausanne University Hospital (CHUV), a tertiary care facility. Polytrauma was defined as an Injury Severity Score (ISS) ≥ 16, in accordance with the criteria established by the German Society for Trauma Surgery [17]. In Switzerland, patients with an Injury Severity Score (ISS) of ≥20 are classified under Highly Specialized Medicine (HSM) [11].

The reuse of clinical data from a previously conducted and recently published institutional series (CER-VD 2016-00928) was approved by the local ethics committee [18]. Ethics committee approval was not required for this cost-reimbursement analysis, as it did not involve patient-level interventions or access to identifiable personal data.

### 2.1. Inclusion and Exclusion Criteria

The study included all consecutive patients involved in road traffic crashes (RTCs) who were admitted via the emergency department between 1 January 2012, and 31 December 2019, with an Injury Severity Score (ISS) ≥ 16 at the time of admission and discharged prior to 1 January 2020. Patients younger than 18 years were excluded.

### 2.2. Data Collection

Data from a recently published single-center retrospective cohort study comparing the performance of three predictive scores for the early identification of clinically significant blunt bowel and mesenteric injuries were reused [18]. The dataset was subsequently expanded to include additional variables such as the Major Diagnostic Category (MDC), the DRG assigned to each case based on the year-specific SwissDRG classification at discharge, the associated CW, and the classification of each hospital stay as either an inlier or an outlier. Inlier cases have a length of stay (LoS) within a statistically defined “normal” national range, and their CW remains unadjusted. Outlier cases, by contrast, fall outside this range and trigger CW adjustments to reflect resource use more accurately. High outlier cases exceed the upper LoS threshold and generate additional reimbursement for each excess day. Low outlier cases are discharged before the lower LoS threshold and lead to deductions. These LoS thresholds represent the national reference range used in the SwissDRG system [2]. Additional data on actual hospital costs, reimbursement amounts, and any supplementary payments were retrieved via queries to the institution’s electronic billing and coding platform (Medstat, Freudiger EDV-Beratung GmbH, Bern, Switzerland). Finally, unless otherwise specified, all monetary amounts reported in this study are expressed in nominal terms. For the econometric analysis, however, all monetary variables were adjusted for inflation and expressed in real terms to ensure consistency over time and avoid bias due to price level changes. The conversion was performed by the authors using the Swiss Consumer Price Index (CPI) published by the Swiss Federal Statistical Office (FSO), based on annual averages and rebased to 2019 = 100 [19]. Each monetary variable (revenue, supplementary payments, costs, and net result) was converted into constant 2019 CHF using the following formula: real value = nominal value/CPI (coding year) × 100. The index values were 100.0 (2012), 99.8 (2013), 99.7 (2014), 98.6 (2015), 98.2 (2016), 98.7 (2017), 99.6 (2018), and 100.0 (2019). As Swiss consumer prices remained relatively stable over the study period, the adjustment had only a marginal effect.

### 2.3. Three Methodological Approaches for Cost-Reimbursement Comparison

#### 2.3.1. The MOOS Method

The first approach analyzed the entire study population (N = 382) as a single unified cohort, reclassified annually according to the SwissDRG tariff versions from 2012, 2013, and 2014, corresponding to the first three years of SwissDRG implementation. This approach was adopted to facilitate a direct comparison with the methodology and findings of the study by Moos et al. [9]. Cost data refer to the treatment year in which care was delivered and were extracted from the institution’s internal cost accounting system. For re-billing under the updated SwissDRG pricing structure, these costs were assumed to remain constant across all years. Reimbursement was calculated using a hospital base rate (BR) of CHF 11,180 per cost weight (CW) point.

#### 2.3.2. The ACTUAL Method

The second approach analyzed the population according to its actual yearly distribution over the eight-year period (2012–2019), with a mean (SD) of 48 (10) patients per year. The analysis of deficit drivers across this extended period leverages a substantial cumulative case volume, which is particularly valuable for investigating financial outcomes in the relatively rare population of patients with severe polytrauma.

Patients were analyzed according to the coding and reimbursement framework applicable at the time of their hospitalization, thereby capturing the real-world context and historical impact of SwissDRG implementation. Cost and reimbursement figures were not calculated retrospectively but directly extracted from the hospital’s analytical accounting system.

This real-world cohort was also used to identify the main determinants of net financial outcomes in polytrauma care. We converted the values in 2019 CHF for a more precise comparability. Given the large number of potential predictors, variable selection techniques were employed to improve model interpretability and mitigate issues of multicollinearity and overfitting. To ensure a robust identification of cost drivers, multiple approaches were applied in parallel.

All analyses were performed using R software (version 4.5.1; R Foundation for Statistical Computing, Vienna, Austria) [20]. LASSO (α = 1), Ridge (α = 0) and Elastic Net (α = 0.5) were estimated with the glmnet package. The penalty parameter λ was selected by 10-fold cross-validation, minimizing the mean squared error. For each method, the “full” model uses the λ that minimizes the cross-validated error (λ_min). The “simplified” model uses the largest λ whose error lies within one standard error of that minimum (λ_1SE), giving a more parsimonious and stable model. Predictors were standardized before penalization, and coefficients are reported on the original scale (2019 CHF).

Categorical variables were entered as binary indicators (0/1). These include sex, each injury mechanism, admission characteristics, clinical scores dichotomized at their published thresholds and in-hospital death. DRG type was entered as two indicators (Type 1, Type 2) with Type 3 as reference. Outlier status was entered as two indicators (low, high) with inliers as reference. Coding year, age, ISS, length of stay and time delays were entered as continuous variables.

For the penalized models, out-of-sample performance was assessed through the cross-validated prediction error used to select λ. The Random Forest (randomForest package, default settings: 500 trees, one third of predictors sampled at each split) was evaluated on out-of-bag observations, on which its permutation-based variable importance is also computed. The stepwise model was selected on in-sample AIC. No independent hold-out sample was used, because the aim was to identify consistent drivers of the deficit rather than to develop a predictive tool.

Firstly, LASSO, Ridge, and Elastic Net, three regularized regression methods, were used to enhance predictive performance while limiting model complexity. LASSO (Least Absolute Shrinkage and Selection Operator) is a type of linear regression that, through a penalty term, shrinks the less relevant coefficients to zero eliminating them from the model, automatically selecting only the most significant variables. Two levels of penalization were considered: one rather strict and another rather permissive, to compare the results [21].

Ridge regression also applies regularization to reduce overfitting. However, unlike LASSO, it retains all variables in the model by shrinking the coefficients of less informative predictors rather than eliminating them. This approach allows for a more comprehensive assessment of the influence of all variables. As with LASSO, two different levels of penalization were applied [21].

The Elastic Net combines the LASSO and Ridge penalties, offering advantages when handling datasets with numerous correlated variables. In this analysis, two distinct levels of penalization were applied [22].

Subsequently, a stepwise regression based on the Akaike Information Criterion (AIC) was performed to iteratively select the subset of variables that most significantly improved the prediction of financial deficit, based on ordinary least squares (OLS) model [23].

Finally, a Random Forest model was employed. This non-linear machine learning algorithm allowed for capturing non-linear relationships that could not be adequately captured by the previously applied linear models [24]. In the Random Forest model, variable importance is indicated by the percent increase in mean squared error (%IncMSE) when a given variable is excluded. A higher %IncMSE reflects a greater contribution of that variable to the model’s predictive accuracy.

Using a database of 26 variables analyzed as potential drivers of financial deficit, the multi-model approach produced a concise, accurate, and interpretable set of key drivers of the financial deficit in polytrauma care (Table 1).

The SwissDRG catalogue is structured into Major Diagnostic Categories (MDCs), which group cases that are clinically similar or have comparable resource consumption into DRGs. Polytrauma-specific DRGs are assigned to a dedicated category, MDC 21A, and are classified as type 1 DRGs in this study. Type 2 DRGs correspond to those related to intensive care, identified by descriptors such as ‘Intensive Care’, ‘Mechanical Ventilation Hours,’ or ‘Intermediate Medical Care Unit (U-IMC).’ DRGs that do not fall under MDC 21A and do not meet the criteria for intensive care are classified as type 3.

The Bowel Injury Prediction Score (BIPS), the Faget score, and the Romain score are clinical tools that integrate physical examination findings, laboratory parameters, imaging features, or a combination thereof, to estimate the likelihood of severe blunt bowel and mesenteric injury (sBBMI) and reduce the risk of delayed diagnosis [18].

#### 2.3.3. The RECODED Method

Finally, the third approach involved a subgroup of 200 patients drawn from the full actual cohort (N = 382), comprising the first and last 100 consecutive cases to form a representative cross-sectional sample. These cases were re-coded and re-grouped according to the SwissDRG classifications and CWs in effect from 2013 to 2023. Retrospective recoding accounts for changes in coding practices over time, including the introduction and discontinuation of codes, thereby enabling consistent longitudinal comparisons and reducing potential bias. The year 2012 was excluded due to the unavailability of the corresponding SwissDRG grouper. This simulation enabled the assessment of temporal trends in reimbursement by applying evolving DRG tariffs to a constant clinical population. It also allowed for an evaluation of how systematic updates in the DRG framework impact hospital revenue for comparable cases, thereby isolating the influence of policy changes from variations in case mix.

A key feature of this method is its integration of updates to medical coding systems over time, including biannual revisions to ICD-10-GM (diagnoses) and annual updates to both the Swiss Classification of Surgical Interventions (CHOP) and the SwissDRG catalog. As in the ‘MOOS’ method, cost data correspond to the actual year of care and were extracted from institutional cost accounting. These costs were held constant when recalculating reimbursements using updated DRG tariffs. Reimbursements were estimated based on a fixed hospital BR of CHF 11,180 per CW point.

## 3. Results

Over the full registry period (2008–2019), 1258 patients were admitted to the CHUV Emergency Department (ED) following a road traffic crash (RTC), of whom 787 were admitted during the period relevant to the present study (2012–2019). Among these, 382 patients (48.5%) had an Injury Severity Score (ISS) ≥ 16, and 273 (34.7%) had an ISS ≥ 20. The most common mechanisms of injury were motor vehicle collisions (254/382, 66.5%), bicycle crashes (72/382, 18.8%), and pedestrian impacts (56/382, 14.7%). Most patients were male (295/382, 77%), with a mean (SD) age of 44 (20) years and a mean ISS of 27 (9.7). The mean (SD) hospital length of stay (LoS) was 17.8 (17.5) days.

Of the 382 patients, 301 (78.8%) were classified as inliers, while 45 (11.8%) were high LoS outliers and 36 (9.4%) were low LoS outliers. Mean (SD) case weight (CW), revenue, and costs were 5.7 (5.8) points, CHF 64,610.7 (65,665.2), and CHF 67,361.5 (67,246.0), respectively, resulting in a mean (SD) financial margin of CHF −2750.8 (30,170.5). Total remuneration for all cases amounted to CHF 24,681,289.3, including CHF 408,410.8 in supplementary payments triggered in 97 cases (25.4%). Total costs reached CHF 25,732,076, yielding an overall net loss of CHF −1,050,782 over the 8-year period.

**Figure 1:**
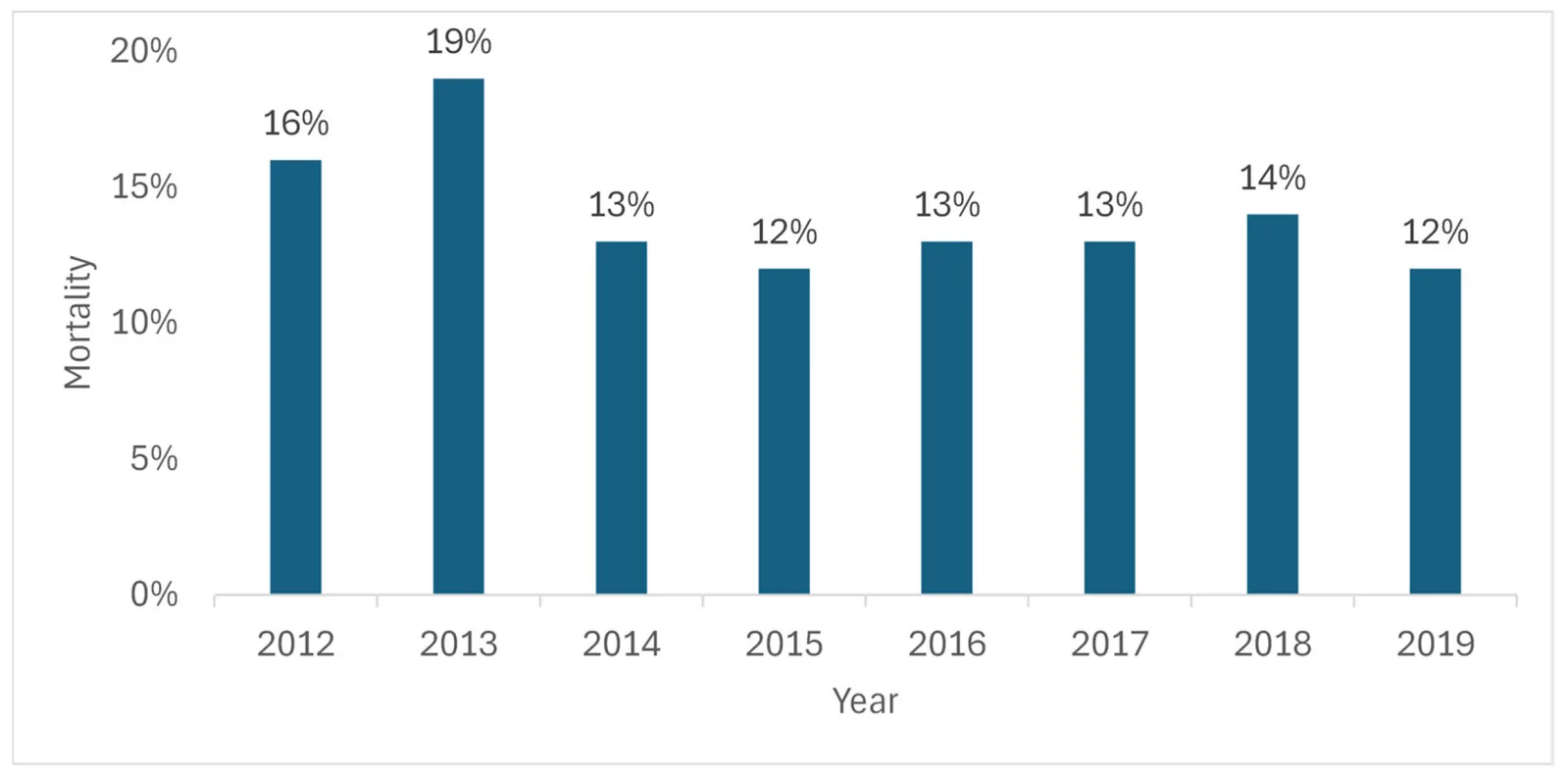
Mortality of patients with ISS ≥ 16 points between 2012 and 2019.

The cumulative case mix (total CW) was 2185.1 points: inlier cases accounted for 1620.2 points; high outlier cases contributed an additional 129.2 points to their base CW of 343.1; and low LoS outliers reduced their base CW of 142.9 by 50.3 points. The overall mortality rate was 16% (61/382), with a median (range) time to death of 1.1 (0–97) days after admission (Figure 1).

In the subpopulation of highly specialized medicine (HSM) patients with an Injury Severity Score (ISS) ≥ 20, the total revenue over the eight-year period amounted to CHF 19.04 million, while total costs reached CHF 20.03 million. This resulted in a cumulative negative margin of nearly CHF 1 million across 275 cases. The mean (SD) revenue per patient was CHF 69,263 (67,409), compared to mean costs of CHF 72,852 (72,242), yielding a mean (SD) deficit of CHF −3588 (32,106) per HSM polytrauma patient.

### 3.1. The MOOS Method

**Figure 2:**
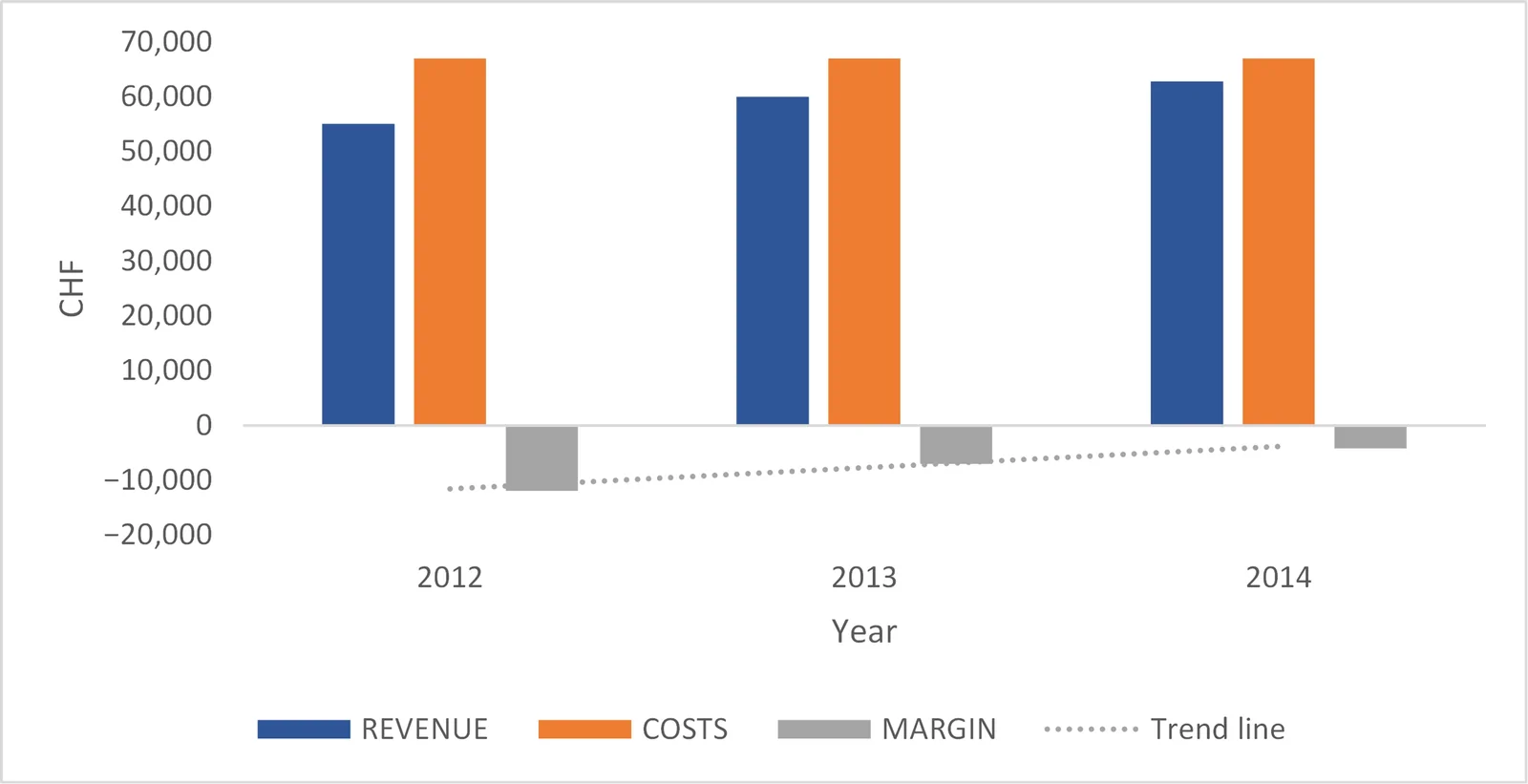
Mean costs, revenue and margin for 382 polytrauma cases per year between 2012 and 2014.

When considering the 382 cases as representative of the annual patient population from 2012 to 2014, the results closely mirror those reported by Moos et al., with a case mix index (average cost weight per case) showing mean complexity over five times higher than the Swiss average [9]. The mean financial margin per patient was markedly negative in 2012 (CHF −12,520.5), and progressively less so in 2013 (CHF −7917.6) and 2014 (CHF −3034.9). Assuming constant mean costs per case (CHF 67,361.5 ± 67,246), Figure 2 illustrates a steady increase in reimbursement amounts over the first three years following the nationwide implementation of SwissDRG: CHF 55,009.1 in 2012, CHF 59,418.6 in 2013, and CHF 63,594.7 in 2014. The trend suggests a trajectory toward full cost coverage by 2015.

### 3.2. The ACTUAL Method

Analyzing the real population per year, the reality shows a different trend, as detailed in Table 2.

**Figure 3:**
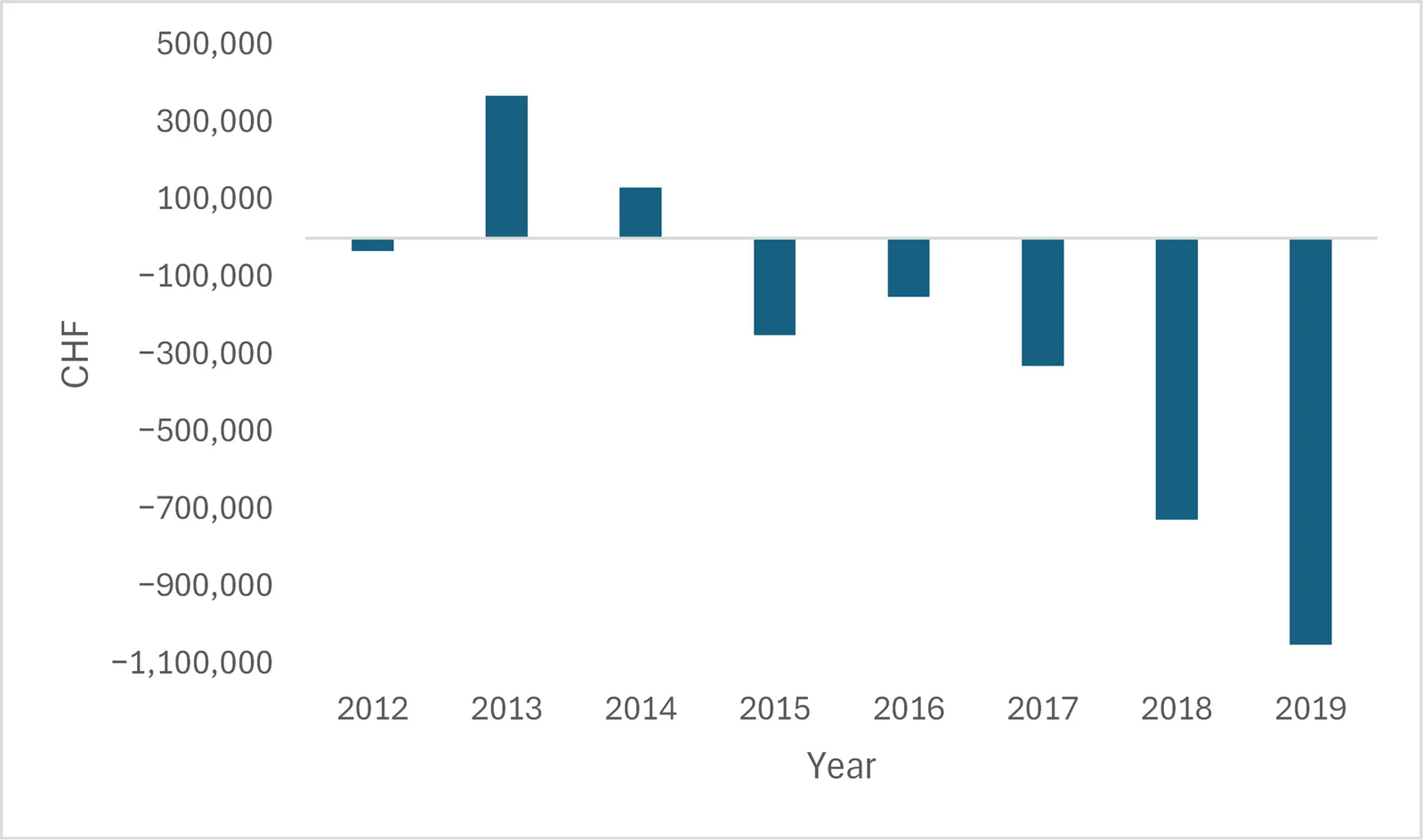
Actual cumulative margin over the 8-yaer period (2012–2019).

Over the 8-year period, total revenue was CHF 24,681,289.3 and total costs CHF 25,732,076.4, leading to a cumulative margin of CHF −1,050,787.1 (Figure 3).

**Figure 4:**
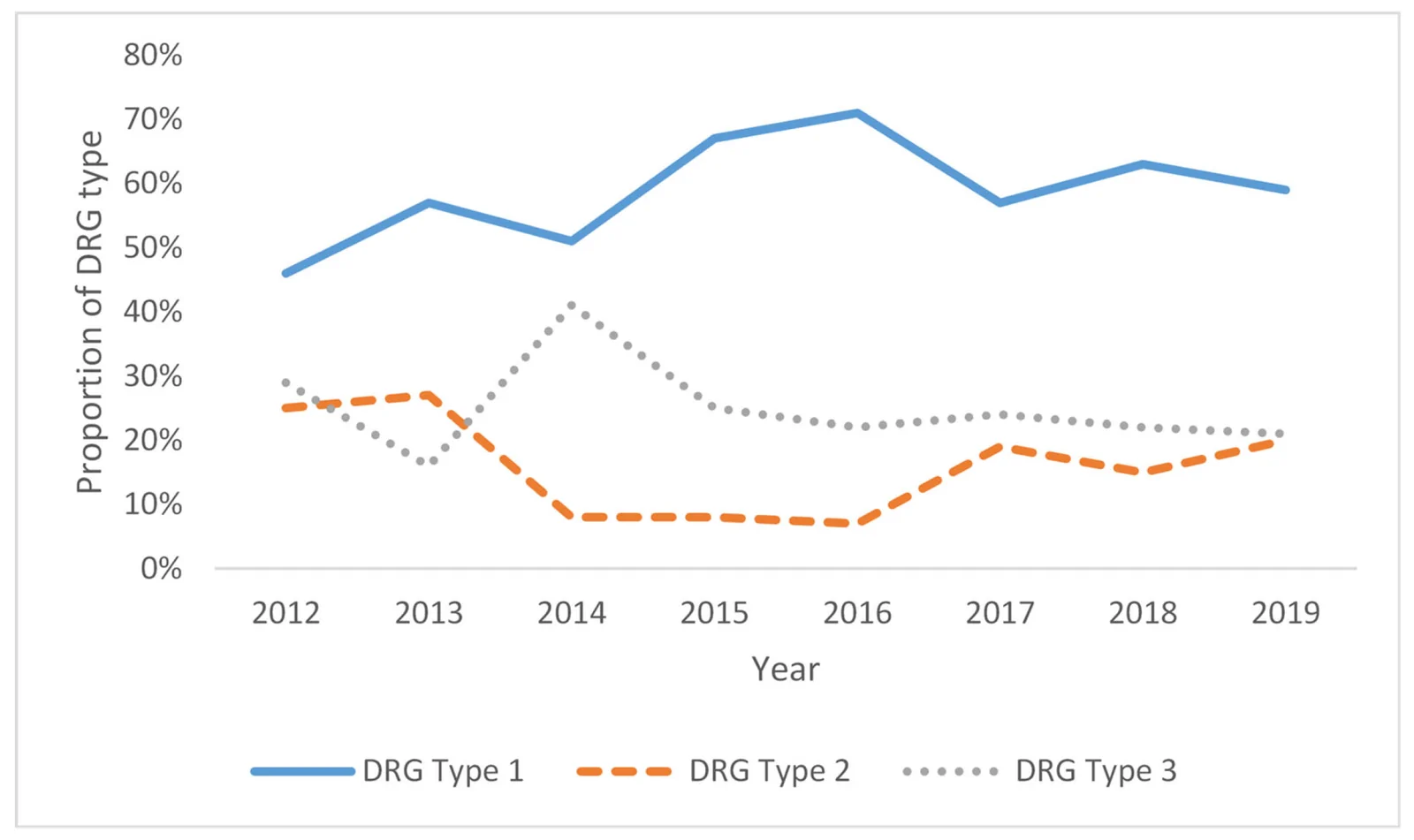
DRG evolution by Type over the 8-year period (2012–2019). DRG Type 1: Polytrauma-specific DRG; DRG Type 2: intensive care unit (ICU)-related DRG; DRG Type 3: Other DRG.

Over time, more cases tended to be classified in a polytrauma-specific DRG (DRG Type 1) (Figure 4).

Over the eight-year period, DRG Type 1 (polytrauma-specific), Type 2 (intensive care unit (ICU)-related), and Type 3 (other) generated mean (SD) financial margins of CHF −2750.8 (30,170.5), CHF −2642.4, and CHF −2688.2, respectively. Type 3 DRGs exhibited the highest proportion of outliers within their group (26.8%), comprising 15.5% low length-of-stay outliers and 11.3% high length-of-stay outliers. In comparison, Type 1 and Type 2 DRGs had outlier rates of 18.6% and 20.3%, respectively.

High length-of-stay (LoS) outliers were associated with a total financial loss of CHF 874,506.2, corresponding to a mean (SD) negative margin of CHF −19,875 (41,438) per patient, responsible for more than 83% of the negative cumulative margin. In contrast, low LoS outliers generated a total positive margin of CHF 43,665, with a mean (SD) margin of CHF 1212.9 (16,488.9) per patient.

#### Main Drivers of Net Financial Results

Among the cost drivers, DRG Type 2 (ICU-related) emerged as the dominant predictor across all regression models (ranging from +18,350 to +30,344 CHF), showing also the highest variable importance in the Random Forest model (%IncMSE = 14.01), indicating a marked association with increased financial deficits. DRG Type 1 (polytrauma-specific) was also significantly associated with higher deficits in the regression models (ranging from +3506 to +7836 CHF) and had a substantial impact in the Random Forest model (%IncMSE = 6.26). The pedestrian hit variable also shows a positive association with increased financial deficits, with the magnitude varying with the model specification. Variables related to patient mortality (in-hospital death and delay between admission and death) were also positively correlated with increased deficits and demonstrated strong relevance in both the regression models and the Random Forest.

Conversely, the variable for high LoS outliers consistently shows a negative correlation with the financial deficit, suggesting that patients with longer hospital stays may reduce the deficit. Other factors that seem to contribute to deficit reduction include a high Romain score and BIPS, being female, long-bone fractures, and longer lengths of stay (LoS).

All variables included in the analysis are presented in Table 3 within their respective models. In the regression models, the coefficients (β) represent the estimated marginal effect of each independent variable on the dependent variable, holding all other variables constant. These coefficients are expressed in 2019 CHF, allowing for direct interpretation in monetary terms. The simplified versions of the regularized models (LASSO, Ridge, and Elastic Net) include only the most robust predictors, selected through more stringent penalization. As a result, their coefficients tend to be smaller in magnitude but more stable.

### 3.3. The RECODED Method

**Figure 5:**
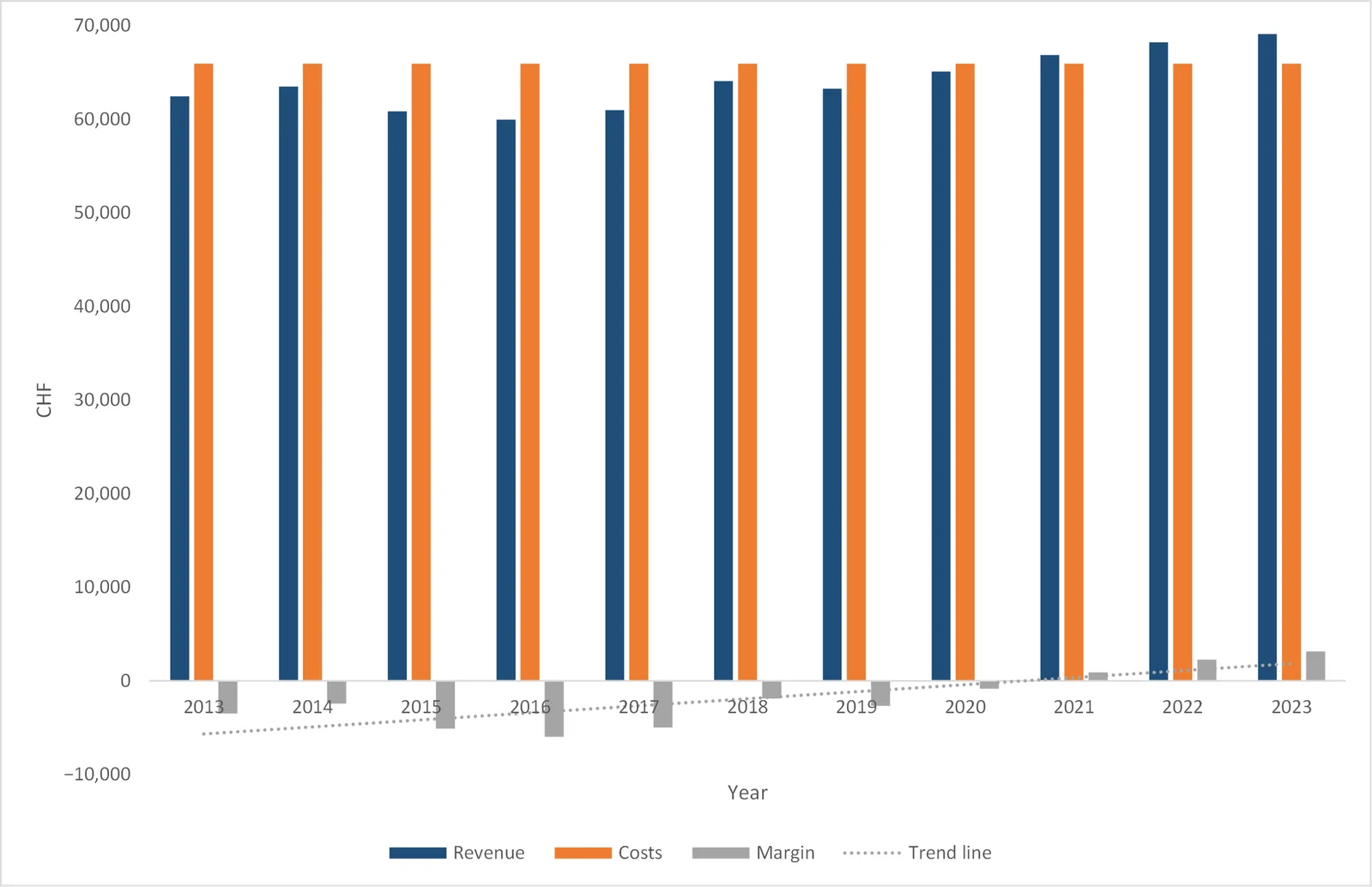
Mean hospital revenue (reimbursement), costs, and margins per patient over an 11-year period (2013–2023).

The current mean (SD) hospital costs remained stable at CHF 65,939 (63,923). The annual evolution of hospital reimbursements (revenues) is illustrated in Figure 5. Contrary to expectations based on the findings of Moos et al. [9], full cost coverage was not achieved in 2015 but only in 2021.

**Figure 6:**
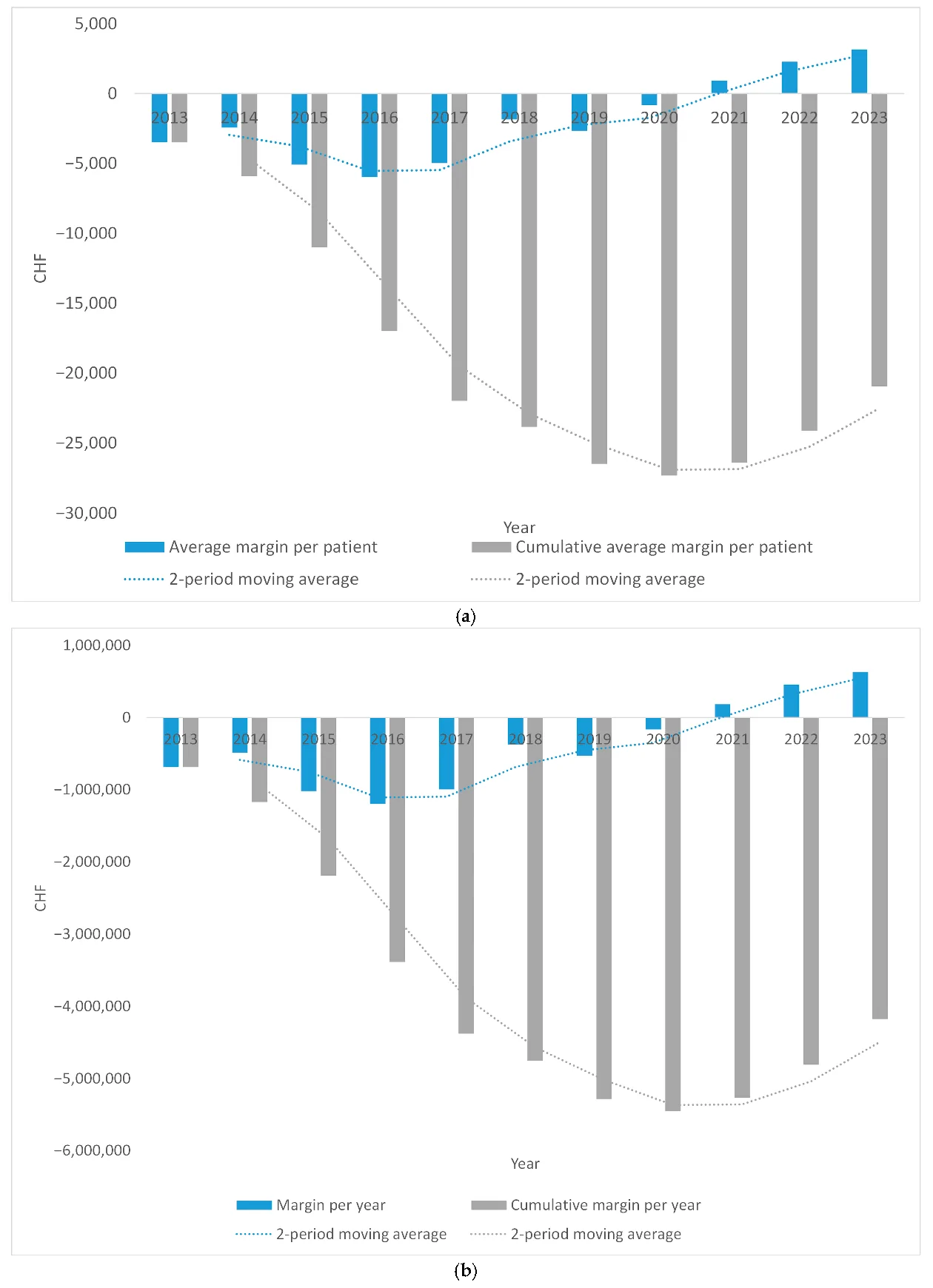
Mean margin and cumulative mean margin per patient and per year: (**a**) mean margin and cumulative mean margin per patient for the 2013–2023 period. (**b**) Mean margin and cumulative deficit per year for the hospital (2013–2023).

Although full-cost coverage was achieved in 2021 using this sample of 200 patients per year, the cumulative losses incurred by the hospital remained substantial (Figure 6). This accumulated deficit over time will only be offset if positive margins continue for a sufficient period, eventually returning to break-even (revenue equal costs) more than 20 years after the implementation of the PPS in 2012.

Throughout the 11-year period analyzed in the RECODED method, the distribution of DRG Types remained remarkably stable, with Type 1 (polytrauma-specific) accounting for a mean (SD) of 60% (2.1), Type 2 (ICU- related) for 18% (1.4), and Type 3 (other) for 23% (0.9) of cases. This consistency suggests that structural DRG allocation has not shifted significantly over time despite changes in coding standards.

## 4. Discussion

The financial sustainability of trauma care remains a pressing concern, particularly in the management of polytrauma patients, which is both resource-intensive and highly variable. With the global adoption of prospective payment systems (PPSs) such as SwissDRG, hospitals face increasing pressure to maintain high standards of care within constrained financial frameworks. This study provides a comprehensive assessment of the net financial losses incurred in the treatment of severely injured patients, underscoring the limitations of current reimbursement models in adequately reflecting the complexity and true cost of care provided by a Level I TC. The extended observation period provides a broader perspective on the system’s adaptation cycle, reducing the risk of interpreting short-term fluctuations or locally favorable trends as evidence of sustained improvement. By integrating three complementary methodological approaches—the historical simulation (MOOS method), the real-world cohort analysis (ACTUAL method), and the retrospective recoding using updated DRG versions (RECODING method)—the study quantifies persistent financial deficits over time. It also highlights the transitional challenges associated with implementing a PPS for a relatively rare and heterogeneous patient population, and identifies key cost drivers contributing to ongoing financial imbalances.

Between 2008 and 2019, a total of 1258 RTC patients were admitted to the emergency department of the CHUV, of whom 787 were included in the present study period (2012–2019). Among these, 382 patients (54%) had an Injury Severity Score (ISS) ≥ 16, and 278 (39%) had an ISS ≥ 20. The study population was predominantly young and male, underscoring that severe trauma remains a leading cause of mortality in this demographic [25,26].

Based on this real-world patient population, three distinct medico-economic analytical methods were employed to capture the multidimensional nature of the topic.

First, the MOOS method, which applied a constant cohort of 382 actual patients to the first three years of SwissDRG implementation, demonstrated increasing reimbursement levels and a trend toward cost coverage by 2015. These baseline findings are consistent with those reported by Moos et al. [9].

Moreover, as the referenced study concluded that the treatment of polytrauma patients was reasonably economical based on benchmarked costs, a similar conclusion can be drawn for the present CHUV cohort. The mean cost per patient was CHF 67,362, which is CHF 1761 lower than the average reported in that study [9].

Although the 9.4% mortality rate recently reported for polytrauma patients with an ISS ≥ 16 represents the lowest recorded to date, the 16% mortality observed in the present study compares favorably with earlier reports from the 2012–2019 period [10,27,28].

Second, the ACTUAL method (analyzing the real patient cohort annually from 2012 to 2019) revealed a persistent cumulative deficit of CHF 1.05 million, despite cases being increasingly classified into polytrauma-specific (Type 1) DRGs. Type 3 DRGs accounted for the highest proportion of outliers, affecting slightly more than one-quarter of patients, which supports improved identification and classification into more specific DRGs to reduce outlier rates. In fact, using the ACTUAL method, high LoS outliers were responsible for mor than 83% of the 8-year cumulative deficit, while low LoS outliers resulted in over CHF 0.5 million in missed revenue over the same period. This shortfall was primarily attributable to the adjustment mechanism applied to inlier case weights, which presumes reduced resource utilization and offers limited alternative DRG options. However, as demonstrated in a previous national analysis of enhanced recovery care by our group, such thresholds may inadvertently penalize high-performing hospitals [29]. This is particularly concerning in the context of complex polytrauma care, where the potential for financial compensation through less costly low-LoS cases is significantly diminished.

Lower and upper LoS trim points may have distinct implications for tertiary referral centers. Patients transferred to a referral center after initial stabilization at another hospital may have shorter stays, potentially falling below the lower trim point, where reimbursement reductions, as discussed above, may further penalize the hospital. Conversely, patients with severe polytrauma often require prolonged hospitalization and may exceed the upper trim point. Although supplementary payments may apply to long-stay outliers, they may not fully cover the additional costs associated with prolonged intensive care and complex treatment. Consequently, the current trim-point mechanisms may inadequately account for the resource requirements and referral patterns characteristic of specialized trauma centers.

Multivariate modeling identified the most significant drivers of financial deficit, partially aligning with the findings of Moos et al. [9]. ICU-related DRGs emerged as the strongest driver, consistently demonstrating the highest coefficients across all linear models, increasing the deficit by amounts ranging from CHF +18,350 to CHF +30,344. This finding was corroborated by the Random Forest algorithm, which ranked ICU-related DRGs as the most influential variable, with the highest importance score of 14.01 (%IncMSE). Notably, the DRG specific to polytrauma was also associated with increased deficits, albeit to a lesser extent. This is particularly concerning, as it indicates that even DRGs explicitly designed for polytrauma do not fully account for the actual costs of care.

Death during the hospital stay, days of delay from admission to death and pedestrian hit are other modest predictors of financial deficits in polytrauma patients under the SwissDRG system. The effects of other variables are less clear, although being admitted at night and each additional year of age slightly increase deficits. Conversely, longer stays tend to reduce deficits, probably because they reflect high outliers that trigger cost weight adjustments, a mechanism adding reimbursement for each day beyond the inlier upper limit for a given DRG [2].

Finally, the RECODED method simulated annual financial performance using a fixed subset of 200 cases, adjusted according to coding standards from 2013 to 2023. Although full cost coverage was achieved in 2021 within this model, cumulative losses remained substantial and would require decades of sustained positive margins to be fully offset. This financial strain appears even more pronounced when focusing on cases meeting the criteria of the HSM mandate, defined by an ISS ≥ 20. Since 2010, these high-acuity cases have been centralized in 12 designated Swiss trauma centers, with reassessment conducted in 2015 [9]. While the average margin for cases with an ISS ≥ 16 was CHF −2750.8 per case, the margin for HSM-designated cases reached CHF −3588 per case. This amplified per-case deficit places an additional financial burden on major trauma centers.

### 4.1. Research Implications

Addressing the financial burden concentrated in a limited number of trauma centers is essential. Although the SwissDRG system is designed to ensure cost coverage in hospitals with a balanced caseload, the centralization of polytrauma care in specialized centers necessitates a more tailored reimbursement strategy at the local level, particularly for cases requiring intensive care. Given the substantial cumulative deficits observed in trauma centers, a gradual approach to adjusting future CWs, rather than pursuing rapid downward revisions, may be a slow but necessary path toward achieving sustainable cost coverage. In parallel, complementary reimbursement mechanisms could help better account for the resource requirements of highly complex polytrauma cases. One option would be to establish a dedicated high-cost outlier pool for patients meeting the criteria for Highly Specialized Medicine (HSM). Supplementary per-diem payments for prolonged ICU stays within MDC 21A could also help bridge the gap between standardized DRG payments and actual treatment costs. Another approach would be to develop separate DRGs for the intensive care and non-ICU phases of hospitalization, allowing the costs associated with each stage of polytrauma management to be more accurately captured and reimbursed. The feasibility, financial impact, and potential unintended incentives associated with these approaches would need to be evaluated in dedicated analyses before implementation.

Additionally, greater awareness of the primary drivers of financial deficits—especially those weighted heavily in the SwissDRG classification—and efforts to improve the categorization of polytrauma patients within ICU-related DRGs are of critical importance. Finally, reevaluating the lower thresholds that define low outliers in polytrauma cases could offer another avenue for improving the fairness and accuracy of reimbursement.

Building on these findings, future research should explore whether similar trends or discrepancies are observed in the prospective payment systems of other countries. Such comparative analyses could help identify structural issues common to case-based reimbursement models and provide valuable insights for targeted adjustments.

### 4.2. Strengths and Limitations

This study provides a long-term, real-world evaluation of inpatient prospective payment for severe polytrauma over more than a decade. The use of three complementary approaches allowed assessment of reimbursement evolution from different perspectives. In addition, combining multivariable modeling with Random Forest analysis enabled identification of key predictors of financial deficits, providing clinically and policy-relevant insights. Nonetheless, this study has several limitations. The RECODED method did not incorporate the 2012 version of the SwissDRG grouper, as this version was no longer available on the SwissDRG SA website at the time of analysis. Furthermore, due to the substantial time and resources required for full recoding, only a subset of patients was included in the RECODED analysis. Although this sampling approach reduced the overall workload, it allowed for an approximate assessment of cost variation across the study period by including cases from both the early and later phases. Moreover, a comparison between the recoded subset (N = 200) and the remaining cohort (N = 182) revealed no statistically significant differences in age, sex, injury severity (ISS), mortality, cost-weight, treatment costs, or reimbursement. These findings provide reassurance regarding the comparability of the two groups for the variables examined.

No sample size or power analysis was performed, as all consecutive patients available during the study period were included. Also, the predictive performance of the models was not validated on an independent sample; the models were used to identify consistent deficit drivers across methods. Additionally, although the use of multiple analytical approaches to assess robustness, considering major collinearities, is praiseworthy, multicollinearity may still affect the models. While this does not necessarily compromise overall model fit or predictive performance, it can obscure the true significance of individual predictors. Furthermore, although a broad range of clinical and administrative variables was considered, the analyses do not establish whether the identified associations persist after simultaneous adjustment for all potentially relevant confounding factors.

The estimated year of break-even was based on an annual sample expanded through the retrospective recoding of 200 cases for each year from 2013 to 2023. No adjustment for the Consumer Price Index (CPI) was applied to the costs, and a fixed base rate of CHF 11,180 was used. Therefore, the estimated year of break-even should be interpreted in the context of these specific assumptions, as alternative approaches to inflation adjustment or different base rates could lead to a different estimate.

Finally, the sample is limited to patients from RTCs, which limits the generalizability of the study findings to the broader population of severely injured patients with other injury mechanisms. Nevertheless, they constitute the predominant source of polytrauma cases at CHUV and in most trauma centers [9,27].

## 5. Conclusions

This study underscores the ongoing challenges to the financial sustainability of trauma care for severely injured patients. Despite ongoing adjustments to the SwissDRG system, reimbursement remains insufficient to fully cover the costs of polytrauma care in tertiary centers. Financial sustainability is particularly threatened by persistent underfunding, especially in high-cost ICU cases. Addressing these challenges requires targeted policy measures, including improved DRG classification for polytrauma patients and particularly for those needing intensive care treatment, consideration of key deficit drivers in future tariff revisions, caution in reducing cost weights, and the introduction of tailored reimbursement models for trauma centers. These reforms are essential to ensure the long-term viability and quality of polytrauma care in Switzerland’s most resource-intensive hospitals, while future research should explore similar issues internationally.

## Figures and Tables

**Table 1:** The 26 variables analyzed as potential drivers for financial deficit.

Domain	Variable
Demographic	Coding year, sex, age.
Trauma characteristics	Collision with a moving object, cyclist, pedestrian hit, light vehicle (car), motorcycle.
Hospital admission	Emergency department (ED) shock room, patient transferred from another hospital, patient intubated at admission, patient admitted during night shift.
Clinical status	Injury severity score (ISS), Faget score ≥ 5 points, BIPS ≥ 2 points, Romain score ≥ 8 points, presence of a long bone fracture.
Reimbursement	DRG Type 1 (specific for polytrauma), DRG Type 2 (intensive care unit (ICU)-related), low LoS outliers, high LoS outliers.
Clinical delays	Delay between ED arrival to computed tomography (CT) scan, delayed diagnosis (>24 h since admission) of a significant blunt bowel and mesenteric injury.
Outcome	Patient’s LoS, patient died during the stay, delay between admission to time of death.

**Table 2:** Mean (SD) results per case in CHF, according to the SwissDRG version applicable each year between 2012 and 2019.

SwissDRG Version (Year)	N Cases	Reimbursement	Costs	Margin
SwissDRG 1.0 (2012)	41	64,583 (59,087)	65,380 (60,166)	−797 (42,078)
SwissDRG 2.0 (2013)	44	80,734 (77,637)	71,632 (70,214)	9102 (38,173)
SwissDRG 3.0 (2014)	39	68,619 (66,981)	74,705 (69,003)	−6086 (29,149)
SwissDRG 4.0 (2015)	36	68,866 (56,327)	79,443 (75,507)	−10,577 (31,168)
SwissDRG 5.0 (2016)	47	59,728 (63,702)	57,626 (54,113)	2102 (26,450)
SwissDRG 6.0 (2017)	53	65,754 (70,265)	69,128 (67,908)	−3374 (26,476)
SwissDRG 7.0 (2018)	54	56,356 (69,940)	63,729 (77,502)	−7373 (19,389)
SwissDRG 8.0 (2019)	68	58,682 (59,833)	63,421 (64,424)	−4739 (26,186)

**Table 3:** Main drivers for net financial result: key findings by models.

	Models Utilized: Unless for RF Model Where %IncMSE ^†^ Is Expressed, All Other Models Express β-Value ^•^, Which Corresponds to the Impact in CHF on the Financial Result							
Variable	LASSO	LASSO Simple ^‡^	Ridge	Ridge Simple ^‡^	Elastic Net	Elastic Net Simple ^‡^	Stepwise LM	RF (%IncMSE)
Coding year	−858		−760	−94	−828		−1106	4.94
Female	−3443		−4477	−343	−3404			0.39
Age	145		140	21	143		152 *	4.52
Cyclist	−104		−1662	126	−105			−1.93
Pedestrian hit	2010		2881	720	2106			1.95
Collision with moving object			725	28				−0.28
Light vehicle (car)			352	−201				2.21
Motorcycle			−875	−312				2.71
ED shock room admission	−2577		−3488	−289	−2461			1.63
Transferred from another hospital			−222	228				−1.59
Intubated at admission	−1351		−1978	184	−1294			3.65
Managed during night shift	1282		1635	18	1206			2.67
ISS (EEP)	−70		−139	−13	−73			3.66
Faget score ≥ 5			495	−212				−0.01
BIPS ≥ 2	−2161		−3158	−727	−2109			0.02
Romain score ≥ 8	−3475		−3994	−873	−3410		−6470	0.20
Long bone fracture	−2209		−3333	−740	−2276			2.26
DRG Type 1	5711		3506	−278	5239		7836 *	6.26
DRG Type 2	26,027	8321	18,350	2067	25,008	8458	30,344 ***	14.01
Low LoS outliers			−68	312				−1.19
High LoS outliers	−9475	−3663	−10,681	−1802	−9731	−3947	−8869	8.62
Delay in arrival to CT scan			−1.5	−0.7				0.85
Delayed diagnoses > 24 h (LMI)			−716	−1 377				0.26
LoS in days (EED)	−314	−30	−194	−26.7	−297	−49	−408 **	7.82
Died during the hospital stay	2249		5665	1203	2552			7.6
Delay in admission to death	951	174	745	104	925	229	1082 **	10.26

**^•^** β-value: Positive β increase deficit (reduce profit or increase loss), negative β reduce deficit (increase profit or reduce loss); ^†^ %IncMSE: Percent increase in mean squared error; ^‡^ simplified model. For the stepwise linear model (LM): *** *p*-value ≤ 0.001, ** 0.001 < *p*-value < 0.01, * 0.01 < *p* < 0.05, (blank) *p* > 0.05 not statistically significant; EEP: Every Extra Point; EED: Every Extra Day; LASSO: Least Absolute Shrinkage and Selection Operator; LM: Linear Model; IncMSE: Percent increase in Mean Squared Error.

## Data Availability

The data presented in this study are available on request from the corresponding author. The data are not publicly available due to privacy and ethical restrictions.

## Abbreviations

The following abbreviations are used in this manuscript:

AIC	Akaike Information Criterion
BIPS	Bowel Injury Prediction Score
BR	Base Rate
CHF	Swiss Francs
CHOP	Swiss Classification of Surgical Intervention
CW	Cost Weight
DRG	Diagnoses-Related Group
ED	Emergency Department
FSO	Federal Statistical Office
HSM	Highly Specialized Medicine
ICU	Intensive Care Unit
ICD-10-GM	International Classification of Diseases, 10th version, German modification
ISS	Injury Severity Score
LASSO	Least Absolute Shrinkage and Selection Operator
LoS	Length of Stay
MDC	Major Diagnostic Category
OLS	Ordinary Least Squares
PPS	Prospective Payment System
RTC	Road Traffic Crash
sBBMI	Severe Blunt Bowel and Mesenteric Injury
SD	Standard Deviation
TC	Trauma Center
U-IMC	Intermediate Medical Care Unit
